# Parliament and the Professions

**Published:** 1918-11-16

**Authors:** 


### PARLIAMENT AND THE PROFESSIONS.

### Tuberculosis in the Army.

The Financial Secretary to the War Office informed Sir Arthur Fell that officers retiring from the Army, whether for tuberculosis or other causes, are dealt with by the Ministry of Pensions. Officers on the half-pay list receive a special grant of three guineas a week towards the cost of treatment for tuberculosis. Sir E. Cornwall stated that a scheme for the domiciliary visiting of discharged men suffering from tuberculosis has been arranged in consultation with the Local Government Board and the National Health Insurance Commission. Necessary instructions to local authorities will shortly be issued, and the scheme is an extension of the arrangements for visiting and after-care which are already being carried out by local authorities in many areas.

### Sauce for the Goose is also Sauce for the Gander.

Sir George Cave informed Mr. Houston that a provision arranging that Regulation 40D of the Defence of the Realm Act shall include men as well as women, as regards the transmission of venereal diseases, is contained in a Bill which is now under the consideration of a Joint Committee of both Houses. Mr. Houston asked if the Government accepts the adage, "What is sauce for the goose is sauce for the gander," and the Home Secretary replied, "Certainly."

### Medical Administrator of the Royal Air Force.

Major Baird informed Major Terrell that the position of medical administrator of the Royal Air Force has been offered to Colonel Fell, R.A.M.C., one of the conditions being that he shall be guided by the principles laid down by the Watson-Cheyne Advisory Committee.

### Massage Nurses.

General Croft asked the Financial Secretary to the War Office whether there is a shortage of massage nurses in order adequately to treat British soldiers; and whether, in spite of their shortage, the nurses are employed to massage German prisoners. Mr. James Hope, who replied, stated that there is a shortage in military hospitals, but that no women are employed to massage German prisoners, a very small number of male masseurs having necessarily to be employed for severe cases.

General Croft was informed by Mr. Forster that the usual rate of pay of massage nurses is £2 10s. a week, with a uniform allowance of £2 half-yearly. There are also some posts paid at the rate of £3 10s. and £3 a week. The proposals of the new Advisory Council in regard to pay are now under consideration.

### Prince of Wales's Hospital, Marylebone.

Captain Carr-Gomm, in a question to the Under-Secretary of State for War, referred to alleged dissatisfaction with regard to the treatment of officer patients at the Prince of Wales's Hospital, Great Central Hotel, Marylebone. Mr. Macpherson said that he was not aware that dissatisfaction existed. The number of patients is 780, and the number of nurses forty-three, nursing orderlies thirty, and, in addition, there are twenty Voluntary Aid Detachment orderlies. Frequent inspections are made without notice, and from a recent report it appeared that the officers are very well looked after.

### Women Doctors in Military Hospitals.

Sir Philip Magnus, in a question to the Under-Secretary of State for War, raised various points in respect of the status and emoluments of women doctors in military hospitals. Mr. Macpherson said that he had found it to be legally impossible to grant commissions in the Army to women, and that legislation would be necessary. He would be glad to consider the granting of marks of distinction, but, personally, was strongly opposed to the utilisation of the present Army marks of distinction for women, because if a woman has not the qualifications for a commission it is no good camouflaging her with marks of distinction. He would prefer such marks to take the form of those given to the administrators and directors of Queen Mary's Army Auxiliary Corps and the Wrens.

### Army Dental Service.

Mr. Pennefather asked if provision could be made for dental treatment of all soldiers returning from abroad whose teeth have deteriorated during their period of active service, and also for the dental treatment, if desired, of all returned prisoners of war whose teeth have deteriorated during captivity. Mr. Forster said that the suggestion was not free from difficulty, but he would consider it.

### Medical Research Committee.

Sir Edwin Cornwall stated that the total sum expended up to the present time under his sanction, and that of his predecessors, under the National Insurance Act, 1911, by the Medical Research Committee has amounted to about £247,000. Details of this expenditure can be found in the Annual Appropriation Accounts presented each year to Parliament.

### Ministry of Health.

The Minister of Reconstruction, Dr. Addison, moved: "That leave be given to bring in a Bill to establish a Ministry of Health and a Board of Health to exercise in England and Wales and in Scotland, respectively, powers with respect to health and local government; and for purposes connected therewith."

Dr. Addison said that he had had very many consultations with the local authorities of this country, with the medical men, and with representatives of the great insurance organisations, and the measure he proposed, except in some unimportant particulars, represents a common measure of agreement. He said that it is impossible either to prosecute a vigorous health policy or to secure efficient administration of it until the consolidation of those Government authorities which have charge of it had been brought about. The Bill does not provide medical treatment for any individual, nor affect the functions of any local authority of any kind. It is purely concerned with bringing together the responsibilities of the different Government Departments, both in England and Scotland, which are concerned in health matters. The Bill brings together under one Minister the powers and duties of the President of the Local Government Board and the Health Insurance Commission of England and Wales. It brings in, also, the powers and duties of the President of the Board of Education with regard to the health of mothers and infants, the duties of the President of the Council with regard to midwives, and of the Secretary of State for the Home Department with regard to the protection of infant life. Other powers will be taken, but not immediately used, in respect of the medical care of school children and of sick soldiers. The Government accept the principle that all services relating to the treatment of the sick and infirm should not be administered as part of the Poor Law, but should be made a part of the general health services of the country, and that the remaining functions of the Poor-Law authorities should also be transferred to other bodies.

